# A bio-enabled maximally mild layer-by-layer Kapton surface modification approach for the fabrication of all-inkjet-printed flexible electronic devices

**DOI:** 10.1038/srep39909

**Published:** 2016-12-23

**Authors:** Yunnan Fang, Jimmy G. D. Hester, Wenjing Su, Justin H. Chow, Suresh K. Sitaraman, Manos M. Tentzeris

**Affiliations:** 1 School of Materials Science and Engineering, Georgia Institute of Technology, Atlanta, GA 30332-0245, USA; 2 School of Electrical and Computer Engineering, Georgia Institute of Technology, Atlanta, GA 30332-0250, USA; 3 George W. Woodruff School of Mechanical Engineering, Georgia Institute of Technology, Atlanta, GA 30332-0405, USA

## Abstract

A bio-enabled, environmentally-friendly, and maximally mild layer-by-layer approach has been developed to surface modify inherently hydrophobic Kapton HN substrates to allow for great printability of both water- and organic solvent-based inks thus facilitating the full-inkjet-printing of flexible electronic devices. Different from the traditional Kapton surface modification approaches which are structure-compromising and use harsh conditions to target, and oxidize and/or remove part of, the surface polyimide of Kapton, the present Kapton surface modification approach targeted the surface electric charges borne by its additive particles, and was not only the first to utilize environmentally-friendly clinical biomolecules to build up a thin film of protamine-heparin complex on Kapton, but also the first to be conducted under minimally destructive and maximally mild conditions. Besides, for electrically charged ink particles, the present surface modification method can enhance the uniformity of the inkjet-printed films by reducing the “coffee ring effect”. As a proof-of-concept demonstration, reduced graphene oxide-based gas sensors, which were flexible, ultra-lightweight, and miniature-sized, were fully-inkjet-printed on surface modified Kapton HN films and tested for their sensitivity to dimethyl methylphosphonate (a nerve agent simulant). Such fabricated sensors survived a Scotch-tape peel test and were found insensitive to repeated bending to a small 0.5 cm radius.

The fabrication of a typical flexible electronic device normally requires the patterned deposition of the component materials on a flexible substrate, but it is extremely difficult to fabricate such electronic devices using traditional full-surface deposition approaches (such as surface sol-gel process, ALD, spin-coating *etc.*) due to their lack of capability for local patterning. Due to the fact that inkjet printing is rapid and low-cost featuring high spatial resolution and very good repeatability, it has become an excellent technique for the patterned deposition and fabrication of high-throughput, high-volume and low-cost electronic devices. With inkjet printing, the designed patterns of the devices can be directly printed from design onto the substrates without the need for dies, masks, chemical etchants, patterning techniques *etc.*

Inkjet printing robust flexible single- or multi-layered electronics, however, can be challenging, mainly due to the fact that most flexible substrates commonly utilized for inkjet printing feature highly hydrophobic and inert surfaces which inhibit the deposition of functional materials with water-based solutions, suspensions or inkjet inks. The complete fabrication of an entire electronic device, however, often needs the deposition of functional materials with both water- and organic solvent-based solutions, suspensions, or inks on the same substrate surface, which raises the need for surface modification to these substrates to reduce their inherent surface hydrophobicity.

Surface modification to highly hydrophobic substrates to allow for the inkjet printing of water-based inks is also motivated by environmental and safety reasons. Most of the solvents used in organic solvent-based inkjet inks (such as 1-hexanol, cyclopentanone, and cyclohexanone) are volatile organic compounds (VOCs) and emit toxic vapors to the air during printing and especially during drying process. For high speed printing and/or for wide format images, the emission from the VOC-based inks becomes more critical. The air pollution caused by the organic solvent-based inkjet inks can be a very serious heath and safety concern if the printing and drying are perfromed indoors. For these reasons, water-based inks are always preferred to their organic solvent-based counterparts. Various efforts have taken place to develop and commercialize water-based alternatives for common inkjet inks which are traditionally organic solvent-based. For example, while traditional silver nanoparticle and carbon material inks are VOC solvent-based, their water-based counterparts have become commercially available from quite a few companies such as Sigma-Aldrich (St. Louis, MO, USA) and Henkel (Düsseldorf, Germany).

Due to their excellent properties (flexibility, mechanical, chemical and thermal stability *etc*.), Kapton films, which are well known to be made of polyimide polymer, are one of the most commonly used substrates for flexible electronics. These films, however, feature inert and highly hydrophobic surfaces. Also due to the exceptional mechanical, chemical and thermal stability of Kapton films, traditional surface modification methods use harsh conditions to tune their inherent hydrophobicity by oxidizing and/or removing part of their surface polyimide. These traditional methods include laser ablation^1^,^2^,^3^, ion-beam etching^4^,^5^, plasma etching^6^,^7^, UV/ozone treatment^6^,^8^, acid^6^,^9^ and/or base^10^,^11^,^12^,^13^ treatments *etc*. The harsh conditions these methods use not only produce environmentally hazardous and health-threatening by-products or wastes (such as corrosive strong bases and acids, carcinogenic benzene, extremely irritating acrolein and other volatile hydrocarbons) which can be a serious environmental and safety problem when such surface modification is done in large scales and/or conducted indoors, but also compromise the structural integrity and the properties of the Kapton substrates. For example, while treatment with a sodium hydroxide solution is one of the most commonly used traditional methods for Kapton surface modification^10^,^11^,^12^, such a treatment not only produces highly corrosive NaOH waste, but also dissolves some surface polyimide leaving pits in the Kapton surface^14^. When fabricating electronic devices on structurally damaged Kapton films, the defects on such films will not only negatively affect the deposition/inkjet-printing quality of the device components but also weaken the mechanical strength of the resulting devices.

Some types of Kapton films, such as Kapton HN and HA films, have a slip additive or filler incorporated in the polyimide matrix to enhance their mechanical properties^12^. However, the additive in Kapton films has been scarcely reported in the literature. It has been mentioned, but without supporting data, that the additive in Kapton HN films was calcium phosphate dibasic (CaHPO_4_)^14^. As will be shown below, we have demonstrated in this work that the additive in Kapton HN contained crystalline calcium carbonate (CaCO_3_) and one or more phosphorus-containing compounds. Both CaCO_3_ and CaHPO_4_ dissolve in acids, so even though the exact composition of the additive in Kapton HN has never been reported, it is probably safe to conclude that part or all the components of the additive in Kapton HN are vulnerable to acids or acidic solutions. Considering the fact that the polyimide polymer matrix reacts with bases or basic solutions^10^,^11^,^12^,^13^, it can be concluded that any surface modification to Kapton HN films in acidic or basic solutions will compromise, to a greater or lesser extent, the structural integrity of the Kapton HN films and their properties. To minimize this compromise, a neutral pH is desired when surface modifying Kapton HN films in aqueous solutions.

To take full advantage of the properties of Kapton HN films, any surface modification to such films should avoid as much as possible compromising their structural integrity and properties, especially when the substrate films are really thin. Kapton HN films are available in a wide range of thicknesses. For extremely thin Kapton HN films, such as Kapton 30HN (thickness 7.5 μm), 50HN (thickness 12.7 μm) and 75HN (thickness 19.1 μm), it is critical that their surface modification is non- or minimally destructive. In the present work, we have developed a bio-enabled, maximally mild and layer-by-layer method to surface modify Kapton HN substrates to allow for excellent printability for both water-based graphene oxide (GO) inks and organic solvent-based graphene, selector and silver nanoparticle inks, thus facilitating the fabrication, especially the fully-inkjet-printing, of flexible electronic devices. The development of this method was inspired by the *in vivo* antagonizing interaction of two clinically used biological molecules, heparin and protamine. Heparin, which is a highly negatively charged sulfated glycosaminoglycan, is a physiological substance and has been widely used in clinical practice as an anticoagulant since its first clinical use in 1959^15^. Protamine, which is a highly positively charged small protein, is a physiological antagonist and has been clinically used to reverse the anticoagulant effects of heparin by binding to it^16^,^17^. In the present Kapton surface modification process, heparin and protamine were used to build up, in a layer-by-layer fashion, a thin film of protamine-heparin complex on Kapton HN substrates. Unlike the aforementioned traditional structure-compromising surface modification methods which use relatively harsh conditions to target, and oxidize and/or remove part of, surface polyimide in Kapton, our surface modification process targeted the electric charges on the additive particles (which were incorporated in the polyimide matrix) and was conducted in aqueous salt solutions at neutral pH, room temperature and atmospheric pressure. To our knowledge, this Kapton surface modification method was not only the first to utilize environmentally-friendly clinical biomolecules, but also the first to be conducted under minimally destructive and maximally mild conditions (as a result, the structural integrity and properties of the substrates were minimally compromised). As a proof-of-concept demonstration of the applicability of this method, single- and multi-layered reduced graphene oxide (rGO)-based chemiresistive sensors were inkjet-printed on surface modified Kapton HN films and tested with a nerve agent simulant, dimethyl methylphosphonate (DMMP).

## Results

### Characterization of Kapton HN films

**Figure 1:**
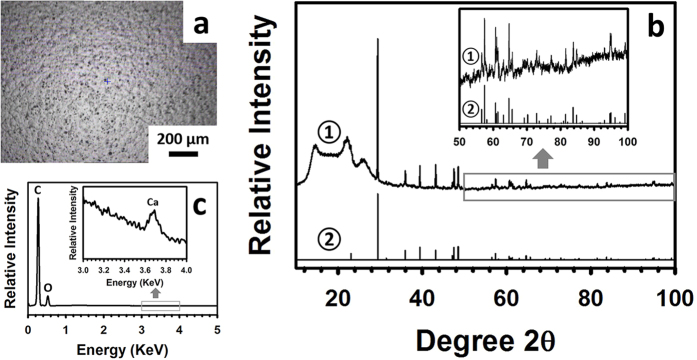
Characterization of Kapton HN films. (**a**) Optical microscopic image of a blank Kapton HN film. (**b**) XRD patterns of the specimen shown in (**a**) (pattern ①) and reference CaCO_3_ (pattern ②, ICDD reference code 04-001-7249) (Inset: Re-scaled XRD pattern to enlarge the area with a 2θ of from 50° to 100°). (**c**) EDX analysis of the specimen shown in (**a**) (Inset: Re-scaled EDX pattern to show the Ca peak).

A number of characterizations were performed on as-received Kapton HN films. Under an optical microscope, numerous particles of varying sizes which were imbedded in Kapton HN polyimide matrix were observed (Fig. 1a). These particles were the slip additive to Kapton HN^14^,^18^. It has been shown that the majority of the slip additive particles exuded to the surface of the substrate^19^, which is consistent with our observation (Fig. 1a). X-ray diffraction (XRD) analysis (Fig. 1b) shows that Kapton HN substrates contained both amorphous (as indicated by the large hump with 2θ ranging from ~10° to ~35°) and crystalline components (as indicated by the sharp narrow peaks). The amorphous component is the polyimide polymer and crystalline component the slip additive. In the past, it has been mentioned that the additive in Kapton HN films was calcium phosphate dibasic (CaHPO_4_)^14^. While CaHPO_4_ might be present in the additive, the crystalline peaks generated from our broad-range (2θ ranging from 10° to 100°) XRD scan matched very well with those of CaCO_3_ (ICDD reference code 04-001-7249) (Fig. 1b), but did not match any of the CaHPO_4_ patterns in the literature or in our XRD databases (PDF-4+2014 and Crystallography Open Database (COD)). Energy dispersive X-ray spectroscopy (EDX) analysis on the Kapton substrate shows significant carbon and oxygen peaks and a small calcium peak (Fig. 1c).

To minimize the interference on the characterization of the additive caused by the polyimide polymer, Kapton HN films were pyrolyzed (to remove the organic polymer) and the resulting inorganic ash was characterized with SEM, EDX and XRD analyses. Before the pyrolysis, a thermogravimetric analysis (TGA) was conducted on Kapton HN substrates in order to find an appropriate pyrolyzing temperature. As shown in Supplementary Figure S1a, the onset temperature for significant weight loss for the organic polyimide polymer was about 500 °C (which is consistent with a previous report^20^) and that for the additive was about 590 °C. When the temperature was increased to about 1070 °C, all the Kapton substrates were completely pyrolyzed. A temperature of 800 °C, which was about the median of the starting and ending temperature values for the pyrolysis of the additive, was chosen to produce Kapton HN ash (this temperature has been shown to be enough to pyrolyze all the polyimide polymer moiety in Kapton HN^20^). Figures S1b,c show the optical images of some Kapton HN pieces in a magnesia crucible before and after, respectively, pyrolysis at 800 °C for 2 hours in air. The white ash particles (Supplementary Figure S1c) resulting from the pyrolysis were characterized with SEM, EDX, and XRD analyses. The size of the ash particles seemed to vary significantly, from less than 100 nm to several microns (Figures S2a,b). The large particles might have resulted from the sintering and agglomeration of fine particles^14^. An EDX analysis showed that the ash contained the elements of oxygen, calcium and phosphorus (Supplementary Figure S2c). The XRD pattern of the ash matched that of calcium pyrophosphate Ca_2_P_2_O_7_ (ICDD Reference code 04-009-6231) (Supplementary Figure S2d). Multiple chemical reactions, including the decomposition of CaCO_3_ into CaO and CO_2_^21^, might have taken place during the pyrolyzing process, which was responsible for the disappearance of CaCO_3_ and the presence of Ca_2_P_2_O_7_ after the pyrolysis.

Combining the information obtained from the characterization of both Kapton HN substrates and their ash resulting from pyrolysis, it can be concluded that the additive in Kapton HN was probably a mixture of CaCO_3_ (crystalline) and one or more phosphorus-containing compounds (crystalline or amorphous). If any calcium phosphate compounds were present in the additive as previously reported, they should be either in crystalline form but in a small amount (that is, beyond the detection limit of the diffractometer) or in amorphous form, or both. Even though the exact composition of the additive in Kapton HN is probably proprietary and currently unknown to the public and more characterizations are needed to identify the phosphorus-containing compound(s), crystalline CaCO_3_ is present in the additive in a significant amount. CaCO_3_ has an isoelectric point of 8.2^22^ and bears positive charges under our surface modification conditions. As a result, during the surface modification process the CaCO_3_ particles on the Kapton HN surface facilitated electrostatic binding of the initial heparin (negatively charged) layer. The subsequent building up of protamine-heparin complex on Kapton HN was facilitated by the electrostatic interaction between the oppositely charged heparin and protamine molecules.

### Printability assessment

As the next step in the evaluation of the Kapton surface modification process, two organic solvent-based inkjet inks (an ethanol-based silver nanoparticle ink and a cyclohexanone/terpineol-based graphene ink) and a water-based GO ink were evaluated for their printability on Kapton substrates before and after surface modification.

**Figure 2:**
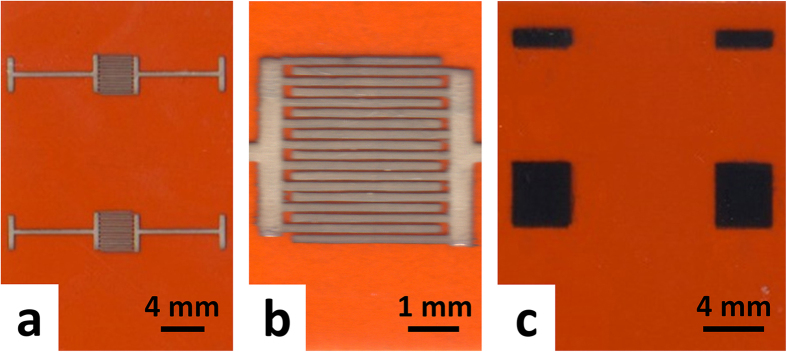
Optical images of proof-of-concept silver and graphene patterns printed on surface modified Kapton HN substrates with an ethanol-based silver ink and a cyclohexanone/terpineol-based graphene ink, respectively. (**a**) and (**b**) Low (**a**) and high (**b**) magnification optical images of the silver interdigitated electrode patterns fabricated by inkjet printing for 5 passes the ethanol-based silver nanoparticle ink on the surface modified Kapton substrates followed by drying at 120 °C for 3 hours. (**c**) Optical image of the graphene square patterns fabricated by inkjet printing the cyclohexanone/terpineol-based graphene ink for 5 passes on the surface modified Kapton substrates followed by drying at 100 °C for 1 hour.

Figures 2a,b show the optical images with low and high magnifications, respectively, of some proof-of-concept interdigitated electrode (IDE) silver patterns inkjet-printed on surface modified Kapton substrates with an ethanol-based silver nanoparticle ink, while Fig. 2c shows the optical image of some proof-of-concept graphene patches inkjet-printed on surface modified Kapton substrates with a cyclohexanone/terpineol-based graphene ink. All these silver and graphene patterns printed with organic solvent-based inks looked morphologically uniform and bore precisely controlled shapes with sharp edges very similar to the designs.

**Figure 3:**
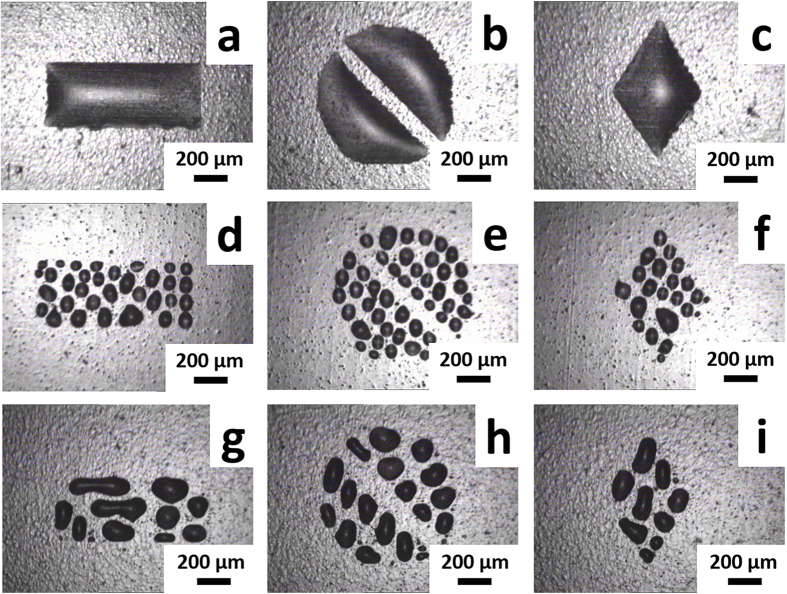
Optical microscopic images of various patterns printed on regularly surface modified Kapton HN (**a–c**), surface unmodified (i.e., blank) Kapton HN (**d–f**), and Kapton HN which had been surface modified with a process in which everything was the same as that in the regular surface modification process (which resulted in patterns **a–c**) except that 1 M NaCl was added to the heparin solution (**g–i**).

As the next evolution step, a water-based GO ink was printed on both blank (surface unmodified) and surface modified Kapton HN substrates. On a Kapton substrate which had been functionalized with the technique reported in this paper, precisely controlled shapes (rectangle, circle with a 100 μm-wide gap in the center, and diamond) were able to be inkjet-printed as designed (Fig. 3a–c). On a blank (surface unmodified) Kapton substrate, however, the GO ink drops made isolated small “islands” (Fig. 3d–f). To verify our hypothesis that the present surface modification method was facilitated by the electric charges on the Kapton HN surface, a control surface modification process, in which everything was the same as that in the regular surface modification process (which resulted in patterns shown in Fig. 3a–c) except that 1 M NaCl was added to the heparin solution (as expected, the pH of the heparin solution was maintained at 7.0 after the addition of NaCl). In the presence of such a high concentration of NaCl at pH 7.0, the positive charges on the Kapton HN substrate surface (which should be due to the positive charges on the surface of the additive, as the surface of the polyimide polymer was uncharged in an aqueous salt solution of pH = 7.0) become screened by the Cl^−^ ions, which should drastically reduce the extent of heparin binding to such modified surface. Indeed, as shown in Fig. 3g–i, the GO ink that was printed on a such modified Kapton substrate balled up and formed isolated small “islands”, similar to the patterns with surface unmodified Kapton HN (Fig. 3d–f), resulting in poor printing quality.

### Fabrication and sensing tests of all-inkjet-printed flexible gas sensors

After the experimental verification that the surface modified Kapton allowed for very good printability for both water- and organic solvent-based inks, various electronic devices were able to be inkjet-printed on the surface-modified substrates. To demonstrate, as a proof of concept, the applicability of the surface modification approach described in this work, multi-layered and single-layered all-inkjet-printed gas sensors were fabricated on surface-modified Kapton HN substrates.

**Figure 4:**
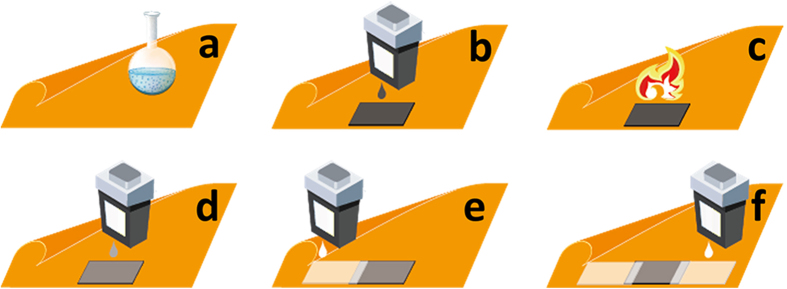
Fabrication of a multi-layered, all-inkjet-printed gas sensor on a surface-modified flexible Kapton HN substrate with both water- and organic solvent-based inks. (**a**) Surface modification of a Kapton HN substrate. (**b**) Inkjet printing of a GO film with a water-based GO ink. (**c**) Thermal reduction of GO into rGO. (**d**) Inkjet printing of a DMF-based selector ink. (**e**) Inkjet printing of one silver electrode with an ethanol-based silver nanoparticle ink. (**f**). Inkjet printing of another silver electrode with the silver nanoparticle ink.

A multi-layered gas sensor was printed following the procedures illustrated in Fig. 4. Briefly, Kapton HN films were surface modified with the process reported in this work (Fig. 4a), and then a GO ink patch was inkjet-printed with a water-based GO ink on the resulting surface-modified Kapton substrate (Fig. 4b). A thermal reduction was performed to convert the electrically non-conductive GO patch into its conductive rGO counterpart (Fig. 4c). A dimethylformamide (DMF) based selector ink made from a hexafluoroisopropanol group-containing compound (2-(2-hydroxy-1, 1, 1, 3, 3, 3-hexafluoropropyl)-1-naphthol) (Supplementary Figure S3a) was then inkjet-printed on the resulting rGO patch (Fig. 4d). Two silver electrodes were finally inkjet-printed with an ethanol-based silver nanoparticle ink on the Kapton substrate, with the resulting electrodes overlapping the rGO patch by 1.5 mm for an optimum contact between the rGO patch and the silver electrodes (Fig. 4e,f). The resulting Ag-rGO-Kapton complex structure was incubated in a 120 °C oven for 3 h for the removal of the organic materials coated on the silver nanoparticles and for the annealing of the silver nanoparticles. A single-layered sensor was similarly fabricated following the procedure illustrated in Fig. 4 except that the selector ink was not printed (i.e., the step illustrated in Fig. 4d was skipped).

**Figure 5:**
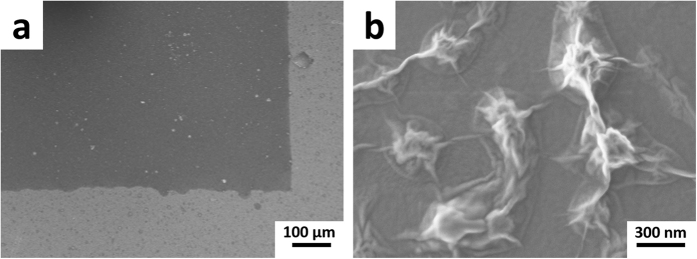
Low (**a**) and high (**b**) magnification SEM images of an inkjet-printed (10 passes) and dried GO film on a surface modified Kapton HN substrate.

In order to examine the micro-/nano-morphology of the inkjet-printed GO patterns, right after the inkjet printing of 10 passes of GO ink (i.e., right after step b in Fig. 4) the resulting GO patch was dried at 95 °C under vacuum overnight to evaporate the water and glycerol and then subjected to SEM analyses. As shown in Fig. 5a (low magnification) and Fig. 5b (high magnification), the dried GO square had sharp edges as designed and the GO flakes were well interconnected with no observable cracks.

**Figure 6:**
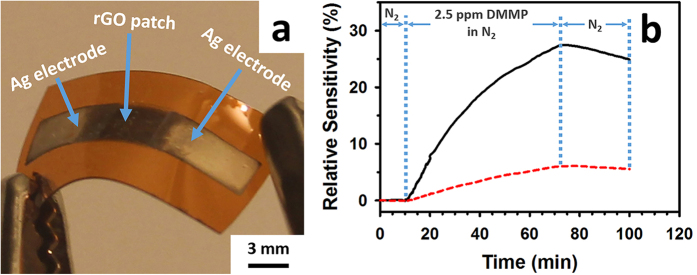
(**a**) Optical image of a fully-inkjet-printed, multi-layered, rGO-based gas sensor. (**b**) Sensing behavior of the multi-layered (black solid line) and the single-layered (red dashed line) gas sensors upon exposure to 2.5 ppm DMMP.

Both the multi-layered and single-layered sensors were flexible, ultra-lightweight (~25 mg), and miniature sized (~1.5 cm × 1.0 cm). Figure 6a shows an optical image of a multi-layered all-inkjet-printed gas sensor prototype (in which the GO ink was printed for 10 passes, the selector ink for one pass, and the silver ink for 5 passes) fabricated following the steps illustrated in Fig. 4. The sensing behavior (relative sensitivity) of the multi-layered and the single-layered sensors upon exposure to 2.5 ppm DMMP (in a N_2_ carrier stream) is shown in Fig. 6b (black solid line for the multi-layered and the red-dashed line for the single-layered). The relative sensitivity S is defined by the formula


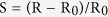


where R is the resistance between the two silver electrodes of a sensor at a particular time after the sensor has been exposed to the DMMP vapor and R_0_ that right before the exposure. For both the multi-layered and the single-layered sensors, the resistance kept increasing upon the onset of 2.5 ppm DMMP. For the multi-layered sensor, it took about 2 min for its relative sensitivity to reach 1% and a 27% relative sensitivity was reached after exposure to the DMMP vapor for 62 min. For the single-layered sensor, it took about 9 min for its relative sensitivity to reach 1% and a 6% relative sensitivity was reached after exposure to the DMMP vapor for 62 min. Upon stopping the DMMP flow, the resistance of both sensors immediately began to decrease slowly (Fig. 6b), indicating the partial desorption the DMMP from the sensing materials.

### Bend cycling testes on all-inkjet-printed flexible gas sensors

A fully inkjet-printed single-layered gas sensor sample was mounted on a universal testing machine with a home-made mounting system. The sample was first bent to a radius of curvature of 1 cm (Supplementary Figure S4a) (with an amplitude of 0.2 cm and a bend rate of 1 mm/second) 1000 times in tension and anther 1000 times in compression. After the 2000 cycles of bending, the resistance of the sensor was found to be virtually the same as that before the bend test (i.e., ~14 kΩ) and no morphological changes were observed with optical analyses. The radius of curvature was then reduced to 0.5 cm (with the amplitude and bend rate remaining 0.2 cm and 1 mm/second, respectively) and the sensor was bent another 1000 times in tension and another 1000 times in compression (Supplementary Figure S4b). The sensor was examined again for its conductivity (with a multimeter) and morphology (with an optical microscope) and again, no apparent conductivity or morphological changes were observed.

### Peel tests on all-inkjet-printed flexible gas sensors

Qualitative Scotch-tape peel tests were performed by sticking the sticky side of a piece of Scotch® magic tape to an inkjet-printed single-layered gas sensor and then peeling the tape off from the sensor^23^,^24^. By visual inspection and optical microscopic analyses, all three components of the sensor (two silver electrodes and one rGO patch), including some small defective areas (small pits caused by scratching) on the silver electrodes, remained intact after the tape had been peeled off (Supplementary Figure S5).

## Discussion

In this work, a bio-inspired, environmentally-friendly and maximally mild process has been developed, for the first time to our knowledge, to surface modify Kapton HN substrates in a layer-by-layer fashion under maximally mild conditions (aqueous salt solutions, neutral pH, room temperature, and atmospheric pressure) which minimally compromised the structural integrity of the substrates yet allowed for the full-inkjet-printing of flexible, lightweight and miniature-sized electronic devices with both water-based and organic solvent-based inkjet inks. Bend cycling tests and peel tests showed that the proof-of-concept gas sensors inkjet-printed on such surface-modified Kapton substrates were mechanically robust. This work has not only introduced a new method to tune the inherent hydrophobicity of Kapton HN films to facilitate the full-inkjet-printing of flexible and robust electronic devices, but also provided a solution for the reduction of the environmental pollution associated with inkjet-printing Kapton-based flexible electronics.

For electrically charged ink particles (such as GO and most metal oxide particles), the present surface modification method has another advantage over traditional Kapton surface modification methods: enhancement of thin film uniformity via reduction of the “coffee ring effect”. The “coffee ring effect”, which originates from the capillary flow in which pinning of the contact line of the drying drop carrying dispersed material(s) ensures that liquid evaporating from the edge is replenished by liquid from the interior^25^, normally occurs during the drying process of inkjet-printed traces and results in the deposition of an appreciable amount of more solid ink material at the film perimeter than the other areas upon ink drying. With the present surface modification method, the target substrate can be modified to bear opposite electric charges of the ink particles by terminating the substrate with either positively charged protamine or negatively heparin. The local electrostatic interactions between the oppositely charged surface-modified substrate and ink particles would inhibit the migration of the ink particles to the film perimeter during drying, thus reducing the “coffee ring effect” and enhancing the uniformity of the inkjet-printed thin films after drying.

As preliminary work for a newly developed Kapton surface modification method, the data reported in this paper are promising but far from comprehensive. More characterizations (such as measurements of contact angles, thin film thicknesses and surface roughnesses as a function of protamine-heparin layers) are needed to better understand and make better use of this method. It is also interesting to investigate and compare the mechanical properties (tensile strength, elongation, shrinkage *etc*.) of Kapton HN films (especially extremely thin films such as HN30, HN50 and HN70 films) before and after surface modification with the technique reported in this work. To the best of our knowledge, mechanical testing on surface modified Kapton films has never been reported.

The difference in sensitivity between the multi-layered (selector-containing) and the single-layered (selector-free) sensors fabricated in this work was probably due to the hydrogen bonding interaction of the analyte DMMP with the hexafluoroisopropanol group-containing selector^26^,^27^,^28^. Compared with the selector-free sensor, the selector-containing sensor probably absorbed more DMMP molecules due to the hydrogen-bonding interaction between the selector 2-(2-hydroxy-1, 1, 1, 3, 3, 3-hexafluoropropyl)-1-naphthol (hydrogen bond donor) and DMMP (hydrogen bond acceptor), making it more sensitive to the analyte. It is worth mentioning that the proof-of-concept sensor prototypes fabricated in this work were not optimized for the analyte. One way to enhance their sensitivity is to use a selector which is more responsive to the analyte. We chose to use 2-(2-hydroxy-1, 1, 1, 3, 3, 3-hexafluoropropyl)-1-naphthol, which is a small and structurally simple compound with only one hexafluoroisopropanol group per molecule (Supplementary Figure S3a), as the selector in this work for our multi-layered sensor due to the factor that it was commercially readily available and inexpensive. We assume that a polymeric compound which contains multiple hexafluoroisopropanol groups per molecule would be more efficient in sensing a G-series nerve agent simulant than the simple selector used in this work since each of such polymeric molecules can absorb more simulant molecules by hydrogen bonding. As a matter of fact, it has been shown that a home-made hexafluoroisopropanol group-containing polycarbosilane polymer, HC (Supplementary Figure S3b), in non-inkjet-printed capacitors^27^ and transistors^29^ was highly responsive to DMMP. Such hexafluoroisopropanol group-containing polymers can be formulated into inkjet inks and applied to our inkjet-printed sensors to enhance their sensitivity to G-series nerve agent simulants such as DMMP.

## Methods

### Surface modification of Kapton HN films

A small Kapton piece with dimensions of 50 mm × 50 mm, cut from a Kapton 500 HN sheet, was first rinsed with 0.2 M phosphate buffer (pH 7.0) and then incubated with a solution of 10 mg/ml heparin sodium in the phosphate buffer (note: the pH of the heparin sulfate solution was 7.0, as confirmed by a pH meter) for 10 min. After rinsing 3 times with the phosphate buffer, the Kapton piece was incubated with a solution of 10 mg/ml protamine sulfate in the phosphate buffer (note: the pH of the protamine sulfate solution was 7.0, as confirmed by a pH meter) for 10 min and then rinsed 3 times with the phosphate buffer. The above process (incubation with heparin, rinse with the phosphate buffer, incubation with protamine, and rinse the phosphate buffer) was repeated 4 times (for a total of 5 times). The surface-modified Kapton substrate was then rinsed with DI water, and dried in air at 60 °C for 2 h.

To validate our hypothesis that our surface modification was made possible by the electric charges on the Kapton HN surface, a control surface modification process, similar to that described above, was performed. During the control process, everything was the same as the process described above except that 1 M NaCl was added to the heparin solution used in each deposition cycle.

### Ink formulation and inkjet printing

A water-based GO ink was prepared by adding an appropriate amount of glycerol to a commercial GO solution to realize a final glycerol concentration of 50 wt %, followed by vortexing to make a homogeneous solution. The resulting GO ink was printed for an appropriate amount of passes on blank (i.e., surface unmodified) and surface modified Kapton substrates. The cyclohexanone/terpineol-based graphene ink was prepared based on the procedures described by Secor *et al*.^30^. The chemoselective compound (selector) ink was prepared by dissolving an appropriate amount of 2-(2-hydroxy-1, 1, 1, 3, 3, 3-hexafluoropropyl)-1-naphthol in dimethylformamide (DMF) to make a solution of 10 mg/ml. The silver patterns were inkjet-printed on the blank and the surface modified Kapton substrates for 5 passes with a commercial silver nanoparticle ink followed by drying at 120 °C for 3 hours. The graphene square patterns were fabricated by inkjet printing the cyclohexanone/terpineol-based graphene ink for 5 passes on surface modified Kapton substrates followed by drying at 100 °C for 1 hour. During the fabrication of the multi-layered and the single-layered sensors, inkjet-printed GO patterns on surface modified Kapton HN substrates were thermally reduced to their rGO counterparts by firing in nitrogen at 300 °C for 1 hour.

All inkjet printing was performed on a drop-on-demand piezoelectric inkjet printer (DMP-2831, Fujifilm Dimatix, Inc., Santa Clara, CA, USA).

### Gas sensing

DMMP vapor stream (2.5 ppm) was generated from a DMMP permeation tube (KIN-TEK Laboratories, Inc., La Marque, TX, USA) in a FlexStream™ Gas Standards Generator (KIN-TEK Laboratories, Inc.) and carried by nitrogen with a flow rate of 500 SCCM. Gas sensing and data recording were performed with a home-made automated sensing system.

### Bend cycling tests

Bend cycling tests were performed on a universal testing machine (TestResources, Inc., Shakopee, MN, USA) controlled by R Controller software. An inkjet-printed single-layered gas sensor sample was mounted on the bend tester with a home-made mounting system. With a bend amplitude of 0.2 cm and a bend rate of 1 mm/second, the sample was first cycled to a minimum radius of curvature of 1 cm 1000 times in tension and another 1000 times in compression, and then cycled to a minimum radius of curvature of 0.5 cm 1000 times in tension and anther 1000 times in compression.

### Peel tests

The adhesion of an inkjet-printed single-layered gas sensor to the surface modified Kapton substrate on which the sensor had been printed was examined via a simple qualitative peel test^23^,^24^. Briefly, the backside of the sensor was glued with epoxy glue (The Gorilla Glue Company, Cincinnati, OH, USA) to a flat plastic surface. With its sticky side facing the front of the gas sensor, a piece of Scotch^®^ magic tape (3 M Company, St. Paul, MN, USA) with a custom-made non-sticky tab was pressed firmly against the sensor. With the non-sticky tab as a handle, the tape was slowly peeled off from the sensor at an angle of ~90° and the sensor was then examined with visual inspection and optical microscopic analyses.

### Materials Characterization

Thermogravimetric analysis (TGA) of Kapton 500 HN was performed on a Netzsch STA 449 F3 Jupiter^®^ simultaneous thermal analyzer (NETZSCH-Gerätebau GmbH, Selb, Germany) with a temperature increase rate of 10 K/min. Scanning electron microscopy (SEM) was conducted with a field emission scanning electron microscope (Leo 1530 FEG SEM, Carl Zeiss SMT Ltd., Cambridge, UK) equipped with an energy dispersive X-ray spectrometer (INCA EDS, Oxford Instruments, Bucks, UK). X-ray diffraction (XRD) analyses were conducted with Cu Kα radiation using a diffractometer (X-Pert Pro Alpha 1, PANalytical, Almelo, The Netherlands) equipped with an incident beam Johannsen monochromator (PANalytical) and an Xcelerator linear detector (PANalytical).

## Additional Information

**How to cite this article**: Fang, Y. *et al*. A bio-enabled maximally mild layer-by-layer Kapton surface modification approach for the fabrication of all-inkjet-printed flexible electronic devices. *Sci. Rep.* **6**, 39909; doi: 10.1038/srep39909 (2016).

**Publisher's note:** Springer Nature remains neutral with regard to jurisdictional claims in published maps and institutional affiliations.

## Supplementary Material

Supplementary Figures

## References

[b1] Least B. T. & Willis D. A. Modification of polyimide wetting properties by laser ablated conical microstructures. Appl. Surf. Sci. 273, 1–11, doi: 10.1016/j.apsusc.2012.12.141 (2013).

[b2] Gallais L., Bergeret E., Wang B., Guerin M. & Benevent E. Ultrafast laser ablation of metal films on flexible substrates. Appl. Phys. A-Mater. 115, 177–188, doi: 10.1007/s00339-013-7901-2 (2014).

[b3] Guo X. D., Dai Y., Gong M., Qu Y. G. & Helseth L. E. Changes in wetting and contact charge transfer by femtosecond laser-ablation of polyimide. Appl. Surf. Sci. 349, 952–956, doi: 10.1016/j.apsusc.2015.05.089 (2015).

[b4] Bachman B. J. & Vasile M. J. Ion-bombardment of polyimide films. J. Vac. Sci. Technol. A 7, 2709–2716, doi: 10.1116/1.575779 (1989).

[b5] Shin J. W., Jeun J. P. & Kang P. H. Surface modification and characterization of N+ion implantation on polyimide film. Macromol. Res. 18, 227–232, doi: 10.1007/s13233-010-0310-x (2010).

[b6] Gouzman I., Girshevitz O., Grossman E., Eliaz N. & Sukenik C. N. Thin film oxide barrier layers: Protection of Kapton from space environment by liquid phase deposition of titanium oxide. ACS Appl. Mater. Inter. 2, 1835–1843, doi: 10.1021/am100113t (2010).

[b7] Inagaki N., Tasaka S. & Hibi K. Surface modification of Kapton film by plasma treatments. J. Polym. Sci. Pol. Chem. 30, 1425–1431, doi: 10.1002/pola.1992.080300722 (1992).

[b8] Le T. R., Lakafosis V., Lin Z. Y., Wong C. P. & Tentzeris M. M. In *2012 IEEE 62nd Electronic Components and Technology Conference (ECTC).* 1003-1008 (IEEE).

[b9] Ghosh I., Konar J. & Bhowmick A. K. Surface properties of chemically modified polyimide films. J. Adhes. Sci. Technol. 11, 877–893, doi: 10.1163/156856197x00967 (1997).

[b10] Huang X. D., Bhangale S. M., Moran P. M., Yakovlev N. L. & Pan J. S. Surface modification studies of Kapton (R) HN polyimide films. Polym. Int. 52, 1064–1069, doi: 10.1002/pi.1143 (2003).

[b11] Thomas R. R., Buchwalter S. L., Buchwalter L. P. & Chao T. H. Organic-chemistry on a polyimide surface. Macromolecules 25, 4559–4568, doi: 10.1021/ma00044a016 (1992).

[b12] Ghosh M. K. & Mittal K. L. Polyimides: Fundamentals and applications. (Marcel Dekker, 1996).

[b13] Park Y. J., Yu D. M., Ahn J. H., Choi J.-H. & Hong Y. T. Surface modification of polyimide films by an ethylenediamine treatment for a flexible copper clad laminate. Macromol. Res. 20, 168–173, doi: 10.1007/s13233-012-0025-2 (2012).

[b14] Williams M. K. . Kapton HN Investigations. 1–16 (MOUND, 1990).

[b15] Best C. H. Preparation of heparin and its use in the first clinical cases. Circulation 19, 79–86, doi: 10.1161/01.CIR.19.1.79 (1959). 13619024

[b16] Rossmann P., Matousovic K. & Horacek V. Protamine-heparin aggregates - their fine-structure, histochemistry, and renal deposition. Virchows Arch. B Cell Pathol. Incl. Mol. Pathol. 40, 81–98, doi: 10.1007/bf02932853 (1982). 6126957

[b17] Constable S., Winstanley P. & Walley T. Medical Pharmacology: A clinical core text for integrated curricula with self assessment. 3rd edn (Elsevier Limited, 2007).

[b18] Wang P. S., Wittberg T. N. & Wolf J. D. A characterization of Kapton polyimide by X-ray photoelectron-spectroscopy and energy dispersive spectroscopy. J. Mater. Sci. 23, 3987–3991, doi: 10.1007/bf01106825 (1988).

[b19] Hin T. Y. Materials and processes to enable polymeric waveguide integration on flexible substrates Ph.D thesis, Loughborough University (2009).

[b20] McClure D. J. Polyimide film as a vacuum coating substrate. Annu. Tech. Conf. Proc.-Soc. Vac. Coaters **53rd**, 608–612 (2010).

[b21] Halikia I., Zoumpoulakis L., Christodoulou E. & Prattis D. Kinetic study of the thermal decomposition of calcium carbonate by isothermal methods of analysis. Eur. J. Miner. Process. Environ. Prot. 1, 89–102 (2001).

[b22] Somasundaran P. & Agar G. E. The zero point of charge of calcite. J. Colloid Interf. Sci. 24, 433–440 (1967).

[b23] Rye R. R. & Ricco A. J. Patterned adhesion of electrolessly deposited copper on poly(tetrafluoroethylene). J. Electrochem. Soc. 140, 1763–1768, doi: 10.1149/1.2221638 (1993).

[b24] Sener U. *Adhesion of copper to UV photo-oxidized Kapton and Upilex-S polyimide surfaces* Master of Science thesis, Rochester Institute of Technology (2004).

[b25] Deegan R. D. . Capillary flow as the cause of ring stains from dried liquid drops. Nature 389, 827–829, doi: 10.1038/39827 (1997).

[b26] Kong L. T. . p-Hexafluoroisopropanol phenyl covalently functionalized single-walled carbon nanotubes for detection of nerve agents. Carbon 48, 1262–1270, doi: 10.1016/j.carbon.2009.11.051 (2010).

[b27] Snow E. S., Perkins F. K., Houser E. J., Badescu S. C. & Reinecke T. L. Chemical detection with a single-walled carbon nanotube capacitor. Science 307, 1942–1945, doi: 10.1126/science.1109128 (2005). 15790850

[b28] Mirica K. A., Azzarelli J. M., Weis J. G., Schnorr J. M. & Swager T. M. Rapid prototyping of carbon-based chemiresistive gas sensors on paper. Proc. Natl. Acad. Sci. USA 110, E3265–E3270, doi: 10.1073/pnas.1307251110 (2013). 23942132 PMC3761578

[b29] Novak J. P. . Nerve agent detection using networks of single-walled carbon nanotubes. Appl. Phys. Lett. 83, 4026–4028, doi: 10.1063/1.1626265 (2003).

[b30] Secor E. B., Prabhumirashi P. L., Puntambekar K., Geier M. L. & Hersam M. C. Inkjet printing of high conductivity, flexible graphene patterns. J. Phys. Chem. Lett. 4, 1347–1351, doi: 10.1021/jz400644c (2013). 26282151

