# Supplementary material for: A bio-enabled maximally mild layer-by-layer Kapton surface modification approach for the fabrication of all-inkjet-printed flexible electronic devices
Source: Sci Rep. 2016 Dec 23;6:39909. doi: 10.1038/srep39909 (PMC5180237; doi:10.1038/srep39909)
Supplement: Supplementary Figures [file srep39909-s1.pdf]

Supplementary Information

A bio-enabled maximally mild layer-by-layer Kapton surface modification approach for the fabrication of all-inkjet-printed flexible electronic devices

Yunnan Fang[1], Jimmy G. D. Hester[2], Wenjing Su[2] Justin H. Chow[3], Suresh K. Sitaraman[3] & Manos M. Tentzeris[2]

[1]School of Materials Science and Engineering, Georgia Institute of Technology, Atlanta, GA 30332-0245, USA

[2]School of Electrical and Computer Engineering, Georgia Institute of Technology, Atlanta, GA 30332-0250, USA

[3]George W. Woodruff School of Mechanical Engineering, Georgia Institute of Technology, Atlanta, GA 30332-0405, USA

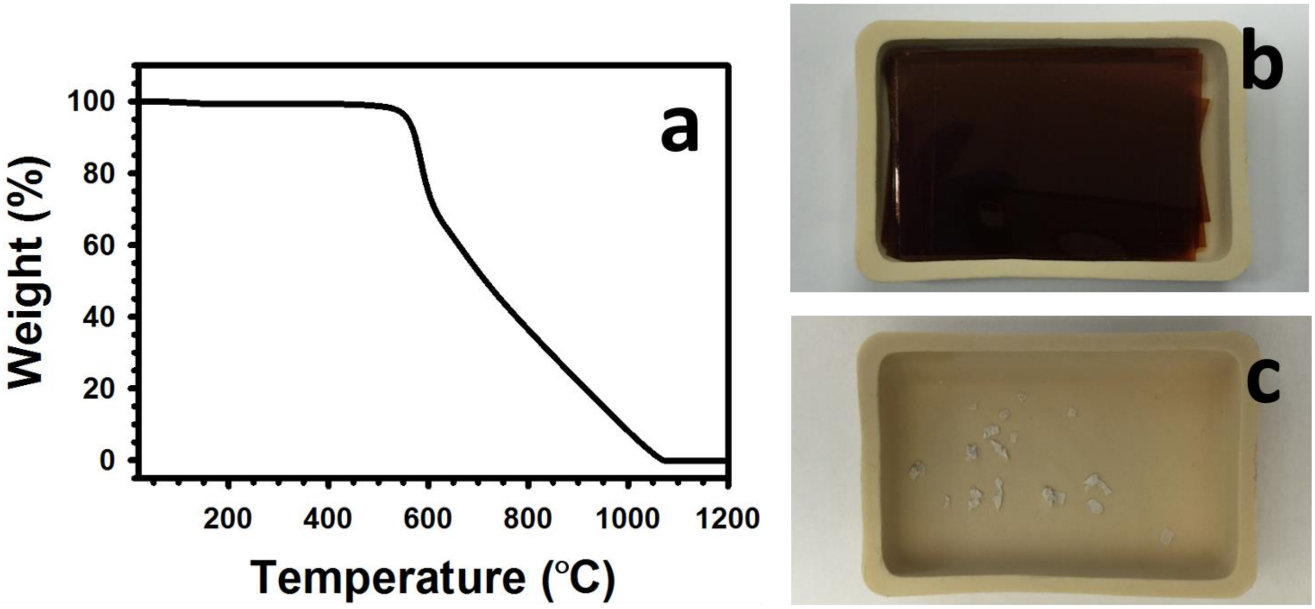

Supplementary Figure S1. Thermogravimetric analysis of Kapton HN films (a) and optical images of the films in a magnesia crucible before (b) and after (c) pyrolysis at 800 °C for 2 hours in air.

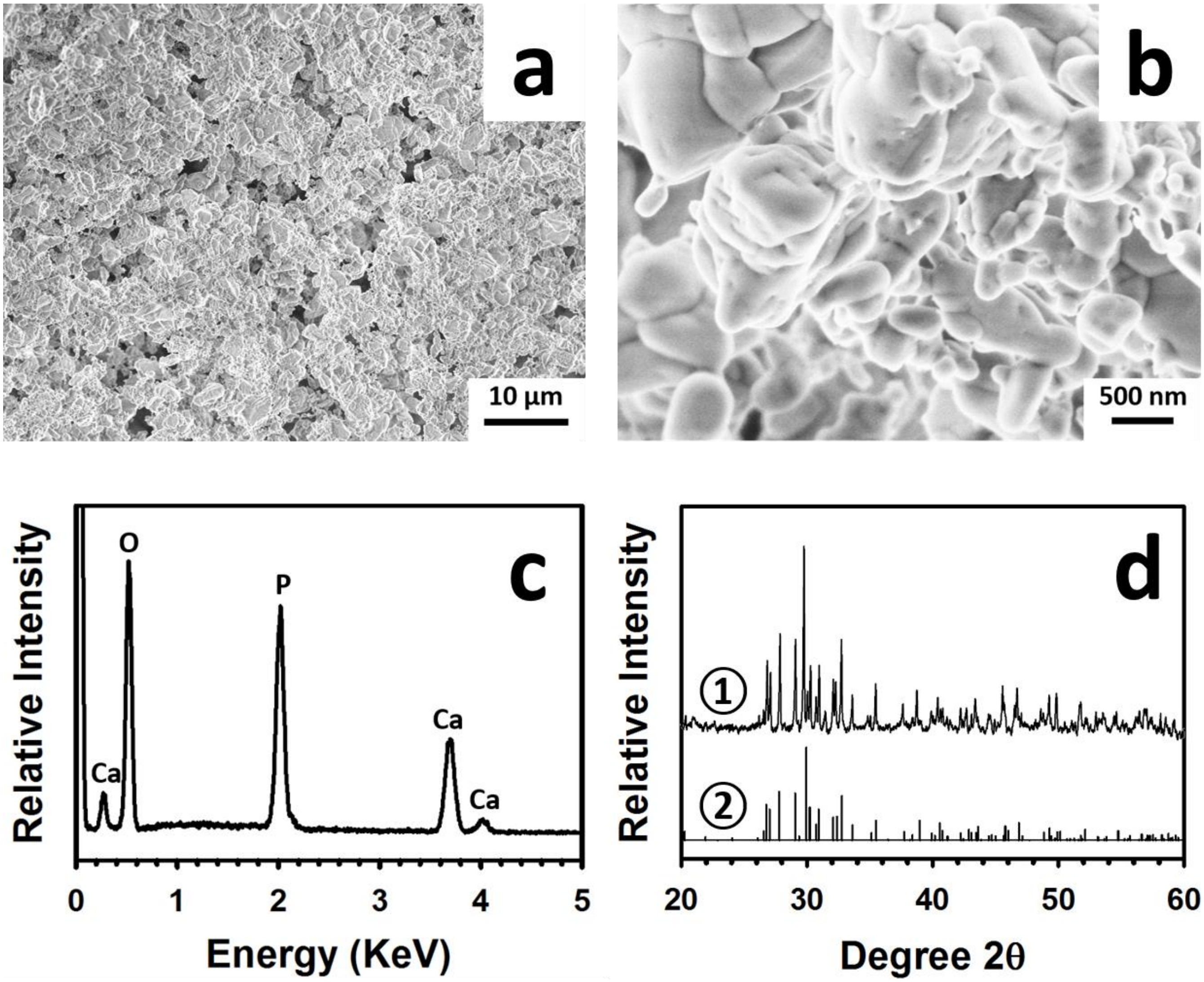

Supplementary Figure S2. Characterization of the Kapton HN ash produced by pyrolyzing the substrate at 800 °C for 2 hours in air. (a) and (b) SEM images with low (a) and high (b) magnification of the ash. (c) EDX pattern of the ash. (d) XRD patterns of the ash (pattern ①) and reference calcium pyrophosphate $Ca_2P_2O_7$ (pattern ②. ICDD reference code 04-009-6231).

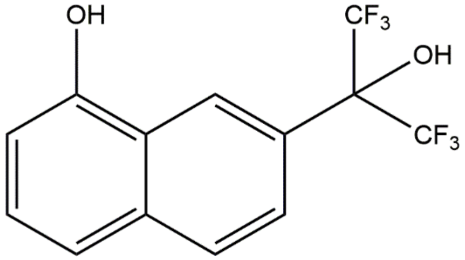

a

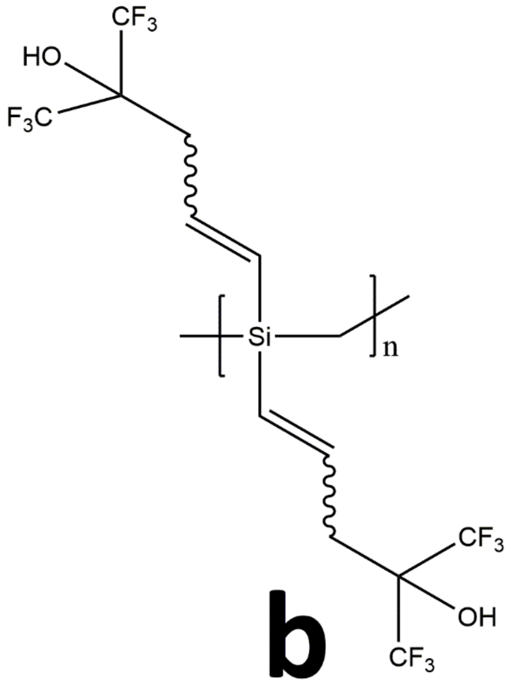

b

Supplementary Figure S3. Chemical structures of two hexafluoroisopropanol group-containing chemoselective compounds. (a) 2-(2-hydroxy-1, 1, 1, 3, 3, 3-hexafluoropropyl)-1-naphthol. (b) HC.

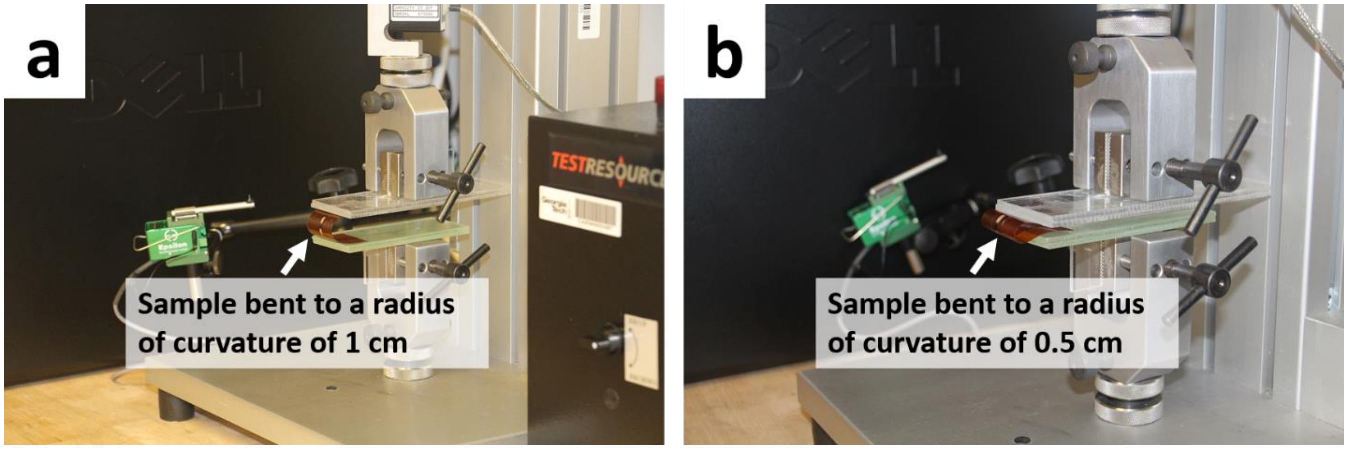

Supplementary Figure S4. A fully inkjet-jet printed gas sensor being bent with a TestResources® bend tester to a radius curvature of 1 cm (a) and 0.5 cm (b).

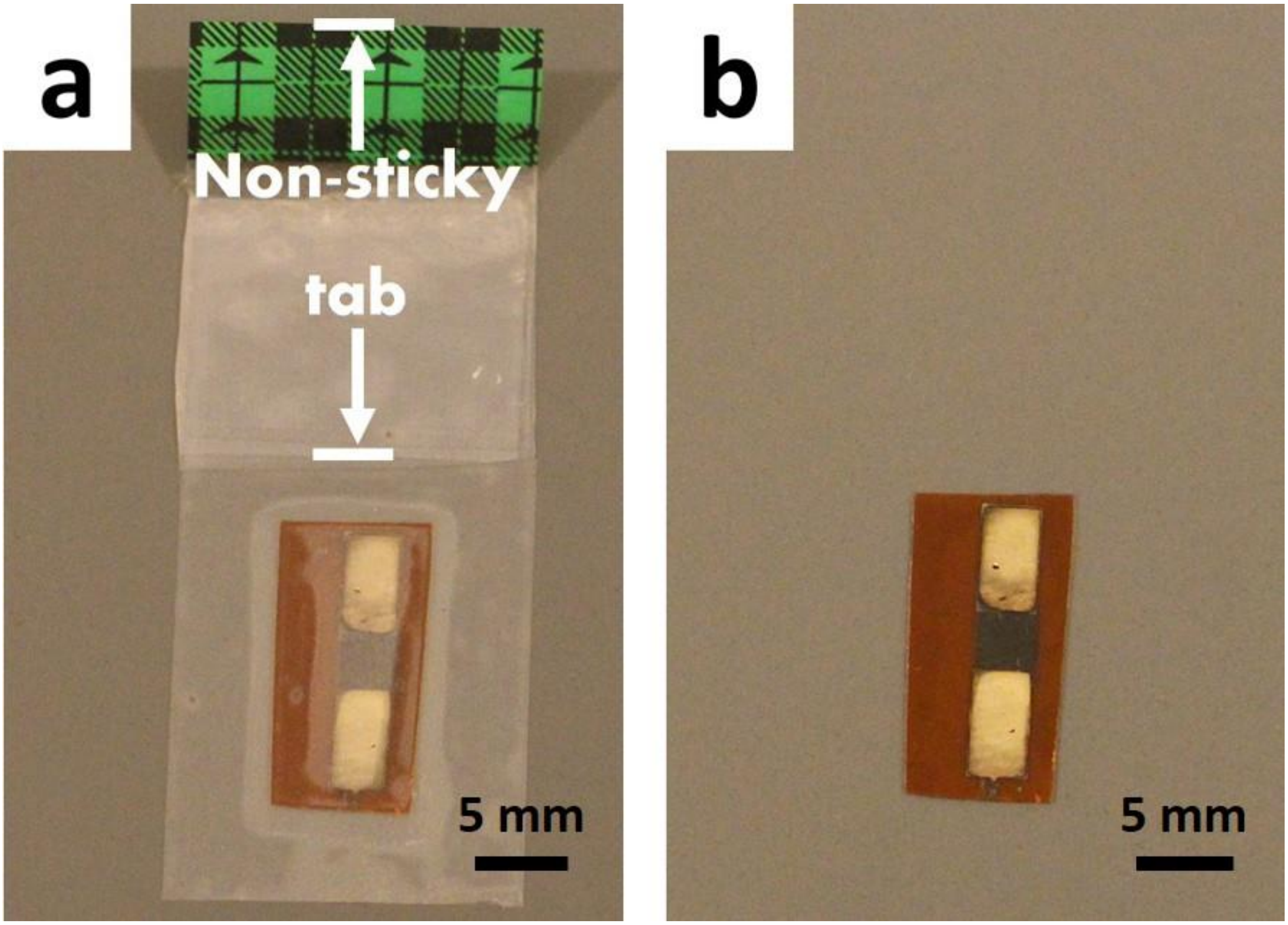

Supplementary Figure S5. Peel test with a fully inkjet-jet printed single-layered gas sensor with Scotch® tape. a). Optical image of the sensor firmly stuck to the sticky side of a piece of Scotch® magic tape with a custom-made non-sticky tab. b). Optical image of the sensor after the peeling off of the tape.
